# Birth weight and breast cancer risk

**DOI:** 10.1038/sj.bjc.6603122

**Published:** 2006-04-25

**Authors:** R Troisi, E E Hatch, L Titus-Ernstoff, J R Palmer, M Hyer, W C Strohsnitter, S J Robboy, R Kaufman, A Herbst, E Adam, R N Hoover

**Affiliations:** 1 Division of Cancer Epidemiology and Genetics, National Cancer Institute, National Institutes of Health, Department of Health and Human Services, Bethesda, MD 20892, USA; 2 Department of Community and Family Medicine, Dartmouth Medical School, Hanover, NH 03755, USA; 3 Department of Epidemiology and Biostatistics, Boston University School of Public Health, Boston, MA 02118, USA; 4 Slone Epidemiology Unit, Boston University, Boston, MA 02215, USA; 5 Information Management Services, Inc., Rockville, MD 20892, USA; 6 Department of Obstetrics and Gynecology, New England Medical Center, Boston, MA 02111, USA; 7 Pathology Department, Duke University Medical Center, Durham, NC 27710, USA; 8 Obstetrics and Gynecology Physicians Organization, Methodist Hospital, Houston, TX 77030, USA; 9 Department of Obstetrics and Gynecology, University of Chicago, Chicago, IL 60637, USA; 10 Department of Molecular Virology and Microbiology, Baylor College of Medicine, Houston, TX 77030, USA

**Keywords:** breast cancer, birth weight, *in utero* exposure, oestrogens

## Abstract

Exploring whether the positive association between birth weight and breast cancer risk differs by other breast cancer risk factors may help inform speculation about biological mechanism. In these data, high birth weight was associated with breast cancer risk in younger and in more educated women, but was not associated overall.

Many, but not all studies of birth weight and subsequent breast cancer risk suggest a positive association, with the most consistent finding being an association in younger or premenopausal women, often with either no or a reduced association among postmenopausal women (Ekbom *et al*, 1992; Michels *et al*, 1996; Sanderson *et al*, 1996; De Stavola *et al*, 2000; Innes *et al*, 2000; Andersson *et al*, 2001; Hilakivi-Clarke *et al*, 2001; Titus-Ernstoff *et al*, 2002; Vatten *et al*, 2002, 2005; Ahlgren *et al*, 2003; Kaijser *et al*, 2003; McCormack *et al*, 2003; Mellemkjær *et al*, 2003; dos Santos Silva *et al*, 2004; Lahmann *et al*, 2004).

We evaluated the association of birth weight and breast cancer risk in the National Cancer Institute's (NCI) Combined Diethylstilbestrol (DES) Cohorts Follow-up Study. The strengths of this resource are the availability of weight from birth records, adult breast cancer risk factor data from three phases of questionnaire follow-up, and a subset of the population receiving very high pharmacologic doses of oestrogen, which could inform some of the speculation about possible hormonal mechanisms.

## MATERIALS AND METHODS

Approvals for the study were obtained from the committees for the review of research involving human subjects at the field centres and the NCI.

The NCI DES Combined Cohort Study started in 1992 with the aggregation of prior US cohorts of individuals with medical record documentation of DES exposure and a comparable cohort of unexposed women (Bibbo *et al*, 1977; Labarthe *et al*, 1978; Greenberg *et al*, 1984). Questionnaires were mailed to participants in 1994, 1997, and 2001, and the National Death Index (NDI)-Plus was used to identify women whose whereabouts were unknown. Of the 5847 eligible subjects with birth weight data who were free of breast cancer at the start of follow-up, 97 developed breast cancer and 1245 were lost before the end of follow-up in 2001; the remaining 4505 were followed through the 2001 data collection phase. Incident cases of breast cancer were identified through questionnaire self-reports and searches of the NDI-Plus. Pathology reports or death certificates were obtained for 91% of the reported breast cancer cases eligible for analysis, confirming invasive disease in 88% and *in situ* disease in an additional 11%. Only primary invasive cases were analysed.

Data on birth weight and gestational age were available from obstetrical charts for 80% of the women. For the remaining 20%, these data were ascertained from the mothers at the time of their daughter's original enrollment in the study (the average age of the daughters**=**24 years). Information on covariates was obtained from the study questionnaires, obstetrical records or interviews, or from earlier questionnaires from the original cohort studies.

Follow-up began on 1 January 1978 (or the date of first enrollment if it occurred later). Person-years accrued until the earliest of the following dates: first breast cancer diagnosis, last known follow-up, death, or return of the 2001 questionnaire. The median number of follow-up years was 23.5 (0.1–25.9 years) for a total of 118 985 person-years.

Poisson regression analysis was used to estimate the age-adjusted incidence rate ratios of breast cancer for each category of birth weight and gestational age. A test for trend was assessed by using an ordinal variable for the birth weight categories. To assess confounding, estimates were individually adjusted for each of the covariates. As a hypothesis-generating exercise, interactions of birth weight with the collected covariates were assessed.

## RESULTS

Birth weight was not associated with attained age, age at first birth/parity, menopausal status, or family history of breast cancer, but was inversely associated with mother's smoking status and use of DES during pregnancy (Table 1). An inverse association between birth weight and age at menarche was also suggested. Birth weight tended to be positively associated with adult height (r=0.25, *P*<0.0001), BMI (*r*=0.03, *P*=0.06), and BMI at age 20 (*r*=0.04, *P*=0.01).

Overall, there was no association between birth weight and breast cancer risk comparing women who weighed <3000 g (rate ratio (RR)=0.93) or >3500 g (RR=1.09) with women who weighed 3000–3499 g at birth (*P* for trend=0.69) (Table 2), and there was no obvious pattern in the association of gestational age with breast cancer incidence (*P* for trend=0.66). These results were similar with simultaneous adjustment for age and gestational age in the birth weight models, or age and birth weight in the gestational age models (data not shown). Estimates changed less than 10% with further adjustment individually for calendar year, and the variables listed in Table 1 (data not shown). With a more detailed examination of birth weight, the RR were 1.3, 0.82, 1.1, and 1.1 for <2500 g, 2500–3000 g, 3500–3999 g, and greater than 4000 g compared with 3000–3499 g.

Among women under the age of 40 years, the RR for women who weighed >3500 g at birth was 2.19 (95% confidence interval (CI) 0.83–5.7) compared with those who weighed 3000–3500 g (Table 3). As the CI indicates, this was an imprecise estimate, based on only 10 cases. High birth weight was associated with an elevated breast cancer risk in highly educated women but a reduction in risk in the less-educated women (*P* for interaction=0.004). However, neither of the estimates was statistically significant and the latter was based on only two exposed cases. There was no evidence of interaction in the association of birth weight with breast cancer incidence by any other breast cancer risk factor, including *in utero* DES exposure, although there were few cases in many of these subanalyses reflected by unstable estimates and wide CIs (data not shown). In analyses restricted to the DES-exposed women, the risk estimates for birth weight and breast cancer by education and age strata were similar to those observed in the combined group of exposed and unexposed women (data not shown).

## DISCUSSION

Most studies find evidence of a positive association between birth weight and breast cancer risk, but several have not (Ekbom *et al*, 1997; Sanderson *et al*, 1998, 2002; Titus-Ernstoff *et al*, 2002; Hodgson *et al*, 2004). Although not associated overall in our data, risk was elevated, albeit not statistically significantly, with high birth weight in younger women consistent with previous observations (Michels *et al*, 1996; Sanderson *et al*, 1996; De Stavola *et al*, 2000; Innes *et al*, 2000; Mellemkjær *et al*, 2003; McCormack *et al*, 2005).

The effect of birth weight varied by level of education with an increased risk for high birth weight in more educated women and an apparent risk reduction in the less-educated women. While earlier studies controlled for social class (Ekbom *et al*, 1997; De Stavola *et al*, 2000; Sanderson *et al*, 2002; Vatten *et al*, 2002, 2005; Titus-Ernstoff *et al*, 2002; McCormack *et al*, 2003, 2005; Lahmann *et al*, 2004; Lahmann *et al*, 2004; dos Santos Silva *et al*, 2004), none found evidence of confounding of the birth weight and breast cancer association. Only one investigated the interaction of birth weight and education (Titus-Ernstoff *et al*, 2002), reporting a stronger association of high birth weight with breast cancer risk in women whose fathers were the most educated. As discussed elsewhere (Hodgson *et al*, 2004), most studies have been conducted in Caucasians from high-risk populations. Results from studies in a relatively disadvantaged population in the US (Hodgson *et al*, 2004) and in Chinese women with limited education (Sanderson *et al*, 2002) suggest an inverse association of birth weight and breast cancer. If the association of birth weight with breast cancer differs by social class, this might explain some of the heterogeneity of findings reported in the literature on birth weight and breast cancer risk. It would be useful to know if any of the other studies with information on socioeconomic status have similar findings.

If the positive association of birth weight and breast cancer risk observed among younger women and those with more education is real and reflects differences in biology, our observation argues against the hypothesis that the operable mechanism is mediated through higher levels of oestrogen. Most of these women (and all in the analyses restricted to DES-exposed women), regardless of their birth weight, received pharmacologic doses of oestrogen during prenatal breast development. Recent observations that cord blood estrogen levels – reflecting fetal exposure – are not associated with birth weight (Troisi *et al*, 2003) also undermine the proposed oestrogen mechanism.

In conclusion, while there was no overall association, we found an elevated risk of breast cancer with high birth weight among younger women and those of higher educational attainment, findings consistent with several other observations. If true, these subgroup differences might explain some of the inconsistencies between existing studies of this relationship. In addition, the presence of the association in our DES-exposed population argues against the popular hypothesis that such a mechanism is oestrogen mediated.

## Figures and Tables

**Table 1:** Distribution of characteristics (person-years (%)) by birth weight category

	**Birth weight (g)**					
	**<3000**		**3000–3499**		**3500+**	
**Characteristic**	**42 054**		**46 398**		**30 533**	
*Cohort*						
DESAD	37 246	(36.1)	39 900	(38.6)	26 000	(25.2)
Dieckmann	4006	(29.3)	5695	(41.7)	3943	(28.8)
WHS offspring	802	(36.5)	803	(36.8)	589	(26.8)
						
*Age (years)*						
<40	28 690	(35.7)	31 027	(38.6)	20 495	(25.5)
40+	13 364	(34.4)	15 371	(39.6)	10 038	(25.8)
						
*Education*						
Some college or less	14 530	(35.2)	15 286	(37.1)	11 412	(27.7)
Completed college	12 853	(35.4)	14 146	(38.9)	9308	(25.6)
Graduate school	10 959	(37.0)	12 002	(40.5)	6643	(22.4)
Missing	3712	(31.3)	4964	(41.9)	3169	(27.6)
						
*Age at menarche (years)*						
<=11	7023	(33.6)	7196	(38.0)	4399	(28.2)
12–13	25 222	(35.3)	28 179	(39.5)	17 907	(25.1)
14+	9599	(37.7)	10 833	(38.6)	8052	(23.6)
Missing	211	(36.6)	190	(33.0)	174	(30.3)
						
*Parity*						
Nulliparous	19 705	(37.0)	20 338	(38.1)	13 212	(24.8)
Age at first birth <30 years	16 005	(33.8)	18 695	(39.5)	12 544	(26.5)
Age at first birth 30+ years	4831	(33.6)	5899	(41.0)	3637	(25.3)
Missing	1513	(36.7)	1466	(35.6)	1139	(27.6)
						
*Menopausal status*						
Premenopausal	35 152	(35.5)	38 500	(38.9)	25 154	(25.4)
Postmenopausal	3063	(34.5)	3533	(39.8)	2261	(25.5)
Unknown/censored	3839	(33.9)	4365	(38.5)	3118	(27.5)
						
*Height (in)*						
<66	27 259	(41.8)	24 940	(38.3)	12 869	(19.7)
66+	13 660	(27.0)	19 993	(39.6)	16 775	(33.2)
Missing	1135	(32.5)	1466	(42.0)	889	(25.4)
						
*Body mass index*						
<25	28 434	(36.3)	30 740	(39.3)	18 972	(24.2)
25+	12 379	(33.4)	14 142	(38.2)	10 488	(28.3)
Missing	1241	(34.5)	1517	(38.8)	1073	(26.5)
						
*Mother's smoking status during pregnancy*						
No	18 546	(29.4)	25 302	(40.1)	19 129	(30.3)
Yes	18 261	(45.9)	14 629	(36.8)	6 833	(17.2)
Missing	5247	(32.2)	6468	(39.7)	4571	(28.0)
						
*DES exposure*						
No	7238	(25.0)	12 234	(42.3)	9395	(32.5)
Yes	34 816	(38.6)	34 164	(37.9)	21 138	(23.4)
						
*Family history of breast cancer*						
No	35 022	(35.8)	37 984	(38.8)	24 680	(25.2)
Yes	4871	(34.1)	5588	(39.1)	3821	(26.7)
Missing	2161	(30.7)	2827	(40.2)	2031	(28.9)

DES=diethylstilbestrol.

**Table 2:** Rate ratios (RR) and 95% confidence intervals (CI) for breast cancer according to birth weight and gestational age

	**No. of cases**	**No. of person-years**	**Age-adjusted RR**	**95% CI**
*Birth weight (g)*				
<3000	32	42 054	0.98	(0.61–1.6)
3000–3499	38	46 399	1.0	—
3500+	27	30 533	1.09	(0.66–1.8)
				
*Gestational age (weeks)*				
<39	21	34 983	0.77	(0.42–1.4)
39	28	23 491	1.38	(0.78–2.4)
40	21	24 246	1.0	—
41+	13	21 772	0.68	(0.34–1.4)
Missing	14	14 493	1.33	(0.67–2.6)

**Table 3:** Rate ratios (RR) and 95% confidence intervals (CI) for birth weight (g) and breast cancer by age, education^a^^,^^b^ and DES exposure

	**No. of cases**	**Age-adjusted RR**	**95% CI**
*Age*<*40 years*			
<3000	7	1.12	(0.39–3.2)
3000–3499	7	1.0	—
3500+	10	2.19	(0.83–5.7)
			
*Age 40*+ *years*			
<3000	25	0.95	(0.56–1.6)
3000–3499	31	1.0	—
3500+	17	0.84	(0.46–1.5)
			
<*4 years of college*			
<3000	16	1.09	(0.55–2.2)
3000–3499	16	1.0	—
3500+	2	0.17	(0.04–0.74)
			
*4 years of college*			
<3000	7	0.75	(0.29–1.9)
3000–3499	11	1.0	—
3500+	9	1.27	(0.52–3.1)
			
*Graduate school*			
<3000	7	1.05	(0.38–2.9)
3000–3499	8	1.0	—
3500+	10	2.27	(0.90–5.8)
			
*DES exposed*			
<3000	15	0.88	(0.51–1.5)
3000–3499	29	1.0	
3500+	18	1.01	(0.56–1.8)
			
*DES unexposed*			
<3000	7	1.32	(0.49–3.5)
3000–3499	9	1.0	1.0
3500+	9	1.36	(0.54–3.4)

a Tests for interaction: *P*=0.22 for age, *P*=0.004 for education, and *P*>0.50 for DES exposure.

b Eleven cases were missing education.

DES=diethylstilbestrol.
